# Seroprevalence of SARS-CoV-2 and Vaccination Coverage among Residents of a Lower-Middle-Class Population in the Federal District, Brazil

**DOI:** 10.3390/vaccines11050916

**Published:** 2023-04-28

**Authors:** Raíssa Nogueira de Brito, Ana Izabel Passarella Teixeira, Carolina Carvalho Gontijo, Rafael Da Silva Faria, Walter Massa Ramalho, Gustavo Adolfo Sierra Romero, Manoel Castro, Vitoria Pessoa, Larissa Araújo Torres, Larissa Pereira Leite, Elza Ferreira Noronha, Rodrigo Haddad, Wildo Navegantes de Araújo

**Affiliations:** 1 Department of Anthropology, University of Georgia, Athens, GA 30602, USA; 2 Campus Paranaíba, Universidade Federal de Mato Grosso do Sul, Paranaiba 79500-000, MS, Brazil; 3 Department of Tropical Medicine, University of Brasilia, Brasilia 70910-900, DF, Brazil; 4 Laboratório de Diagnóstico Molecular, Hospital Universitário de Brasília, Brasilia 70910-900, DF, Brazil

**Keywords:** seroprevalence, COVID-19, vaccination coverage, Brazil

## Abstract

Estimating seroprevalence and vaccination coverage against COVID-19 is crucial to the development of well-targeted public health policies at the local level. Here, we estimated seroprevalence and vaccination coverage in a lower-middle-class population in Brazil. We conducted an observational, cross-sectional, population-based survey from 24 September to 19 December 2021. CMIA tests were used to detect anti-SARS-CoV-2 IgG against the N-protein. The overall seroprevalence was 24.15% (177/733), and vaccination coverage was 91.40% (670/733); 72.09% (483/670) were fully vaccinated. Among vaccinated participants, seroprevalence was 24.77% (95% CI 21.50–28.04; 166/670), with a prevalence ratio (PR) of 1.03 (95% CI 0.98–1.08; *p*-value 0.131). Among participants who received an mRNA vaccine with S-based epitope (485), seroprevalence was 16.29% (95% CI 13.04–19.85; 79/485). Among unvaccinated participants, seroprevalence was 17.46% (95% CI 10.04–28.62; 11/63). Finally, in spite of the political climate and other possible causes for vaccine hesitancy, the positive Brazilian culture towards vaccination might have curbed hesitancy.

## 1. Introduction

The coronavirus disease (COVID-19) is a respiratory illness caused by the severe acute respiratory syndrome coronavirus-2 (SARS-CoV-2). The virus most likely emerged in the Huanan live wildlife market in Wuhan City, Hubei Province, China, in early December 2019 [1], and the World Health Organization (WHO) declared the COVID-19 outbreak a pandemic on 11 March 2020 [2]. To date, more than 600 million SARS-CoV-2 infection cases and 6 million COVID-19-related deaths have been reported in the world. Brazil ranks second in deaths worldwide—behind the United States—and fifth in the number of reported infections, after the United States, India, France, and Germany [3].

Building on pre-existing technologies, the development of different COVID-19 vaccines was accelerated. This resulted in four main types of COVID-19 vaccines that were tested through clinical trials and approved for use across the globe: (1) whole inactivated virus vaccines, (2) protein subunit vaccines, (3) viral vector vaccines, and (4) mRNA (or nucleic acid) vaccines [4]. Except for the SARS-CoV-2 whole inactivated virus vaccine, all others are based on the SARS-CoV-2 viral spike protein (S) for inducing the production of potently neutralizing antibodies. In their turn, inactivated virus vaccines can elicit immune responses that target other viral proteins (e.g., nucleocapsid [N], envelope [E], and matrix [M]) in addition to S [5]. 

Mass vaccination against SARS-CoV-2 significantly reduced COVID-related deaths and reported infections around the world [6]. Nevertheless, the difficulty of vaccine acquisition by countries from the Global South threatens to prolong the pandemic, leading to delays in economic recovery, and, hence, to the exacerbation of inequities. Furthermore, poorly targeted vaccination programs may result in vaccine hesitancy and low vaccination coverage, allowing for the occurrence of genetic mutations and, therefore, the emergence of vaccine-resistant viral strains [7,8]. Whereas vaccination against SARS-CoV-2 began in the United Kingdom by early December and in the United States and Canada by early December 2020 using an mRNA vaccine, vaccination started in Brazil over a month later with the distribution of an imported whole inactivated virus vaccine. Batches of imported viral vector and mRNA vaccines arrived in Brazil only by the end of January and May 2021, respectively. 

In addition to vaccination, past infections have the potential to generate individual protective immunity against SARS-CoV-2. Estimating the number of people infected with SARS-CoV-2 and the vaccination coverage is crucial to understanding the health situation of a given region and to developing new, well-targeted public health policies at the local level (e.g., [9,10]). Here, we estimated the seroprevalence of SARS-CoV-2 and the vaccination coverage among residents of a lower-middle-class population in the Federal District, Brazil through an observational, cross-sectional, population-based survey. Further, we discuss how the adherence to vaccination and the conduction of the vaccination program in Brazil and the Brazilian Federal District relates to our findings.

## 2. Materials and Methods

We conducted a cross-sectional, population-based survey to estimate the seroprevalence of SARS-CoV-2 in the lower-middle class population of São Sebastião (RA XIV São Sebastião), a city in the Federal District of Brazil. The collection of primary data and biological samples was approved by the Research Ethics Committee of the Faculty of Medicine at the University of Brasilia (CEP-FM/UnB, CAEE 39866620.4.0000.5558). All participants were volunteers and signed informed consent forms with research goals, risks, and confidentiality of data prior to data and sample collection. All people with disabilities or under the age of 18 who agreed to participate in this study also had their parents’ or guardians’ consent. This work was conducted in accordance with the Ethical Principles for Medical Research in Human Subjects (Declaration of Helsinki). 

### 2.1. Study Population

São Sebastião (RA XIV, Federal District, Brazil) is a city in Federal District, Brazil, located 22 km from the Brazilian capital, Brasilia. The region was first occupied during the late 50s and is now inhabited by over 115,000 people [11]. According to the official publication [11], sociodemographic data depict a population with mid-to-low monthly income (BRL 1374.50 or USD 270.16 US per capita as of January 2022), low levels of formal education (2.7% are illiterate, 34.6% have completed only elementary school), and a Human Development Index (HDI) of 0.645. It is constituted of both urban and rural areas. The resident population identifies mostly as mixed race/black (64.2%) and white (34.0%), and wealthier areas, such as Mangueiral, are predominantly white (49.3%).

### 2.2. Sample Size

Sample size was calculated as n = [DE × Np(1 − p)]/[d^2^/Z^2^_1 − α/2_ × (N − 1) + p × (1 − p)] in which design effect (DE) was settled as 1, expected prevalence (p) of infection as 19%, confidence limits (d) as 3%, and population size (N) as 115,000 [11]. The expected prevalence (p) used to calculate our sample size was based on our previous study carried out in Cidade Estrutural (RA XXV SCIA/Estrutural), another city in the Federal District, Brazil in 2021, where we found a SARS-CoV-2 seroprevalence of 19% (manuscript in preparation). Thus, our calculated sample size was at least 653 individuals—one person in each selected household.

### 2.3. Study Design and Sampling Strategy

We used the Federal District 2020 shapefile (https://www.geoportal.seduh.df.gov.br/geoportal/ (accessed on 3 January 2022)) to delimit the area of interest for our study. Using the software QGIS 3.10.14 (http://www.qgis.org (accessed on 20 January 2023)), we marked the urban area of São Sebastião as a polygon in the shapefile, excluding industrial sectors, non-residential public buildings, parks, and rural areas. A new shapefile with the marked polygon comprising only the urban area of São Sebastião was created to randomly draw eligible households. We drew 1306 points (653 original samples plus 653 spare samples) over the São Sebastião urban area shapefile. We added a multi-ring buffer to the layer to create a radius of 0.00019 degrees—or 20.3 m. Points that were not located on buildings were manually moved to the nearest building, respecting the radius limit. Sampled points were stored in *.kml files and uploaded into Locus Map 4.5.5—an offline navigation Android application installed on tablets to navigate to eligible households. Five work teams—composed of a driver, an interviewer, and a phlebotomist—visited the sampled points Thursday through Sunday, between 24 September and 19 December 2021. All residents of São Sebastião were eligible to participate in our study. One resident of each drawn household was selected by the work teams using a table of random numbers. Sampled points were replaced by spare points when (i) the eligible household refused to participate, (ii) there was no building within the radius of each point, or (iii) no one was found in the house after three consecutive visits (Supplementary Figure S1). 

### 2.4. Collection of Data and Blood Samples

Primary data and blood samples were collected during household visits after informed consent forms were signed. Data were collected by an interviewer using standardized questionnaires in the data capture software Research Electronic Data Capture (REDCap 9.3.8) [12] hosted at the University of Brasília. Peripheral blood was collected via venipuncture by a licensed professional. Blood samples were stored in two 5 ml tubes containing a coagulation activator and transported at 4–6 °C to the Laboratório de Diagnóstico Molecular (Laboratory of Molecular Diagnosis) at Hospital Universitário de Brasília in Brasília. Data and samples were identified with a serial number corresponding to each participant to ensure their confidentiality and anonymity. 

### 2.5. Serology Tests

The sera obtained from the blood samples by centrifugation were tested with anti-SARS-CoV-2 IgG chemiluminescent microparticle immunoassay (CMIA) (Abbott Laboratories, Sligo, Ireland) using the ARCHITECT PLUS i2000SR system, according to the manufacturer’s instructions. This immunoassay is an automated analysis in which IgG antibodies against SARS-CoV-2 bind to nucleocapsid-antigen-coated microparticles. An acridinium-labeled anti-human IgG antibody conjugate is added to create a reaction mixture, which is then incubated. The resulting chemiluminescent reaction is measured in relative light units (RLU). There is a direct relationship between the amount of IgG against SARS-CoV-2 and RLUs. The ARCHITECT PLUS i2000SR system calculates the average RLU signal from the calibrators. The output is reported as the sample result divided by the store calibrator result. If the test result was <1.4, the sample was considered negative; if it was ≥1.4, the test result was considered positive. Participants with a positive serological test result were considered as previously exposed to SARS-CoV-2, either by vaccination or infection, and participants with a negative serological test result were considered as not previously exposed to the virus (i.e., case and non-case, respectively). 

### 2.6. Statistical Analysis

Serological test results and participant data were tabulated and analyzed using Microsoft Excel 365 and SPSS version 22 [13]. Participants with missing data or unsuitable samples were excluded from the study. We calculated the overall prevalence of immunoglobulin G (IgG) against the nucleocapsid protein (N) of SARS-CoV-2 for our sample, as well as for the vaccinated and unvaccinated groups according to sex, age, race/ethnicity, education level, monthly income, and the number of residents per household. The prevalence ratio (PR) and confidence intervals of 95% (CI 95%) were also calculated for the vaccinated and unvaccinated groups. The attributable fraction was calculated for those participants that were not vaccinated but could have already received at least one shot of the COVID-19 vaccine at the time our survey was carried out.

This study is reported as per the Strengthening the Reporting of Observational Studies in Epidemiology (STROBE) guidelines (Checklist S1).

## 3. Results

### 3.1. Sample Description

**Figure 1:**
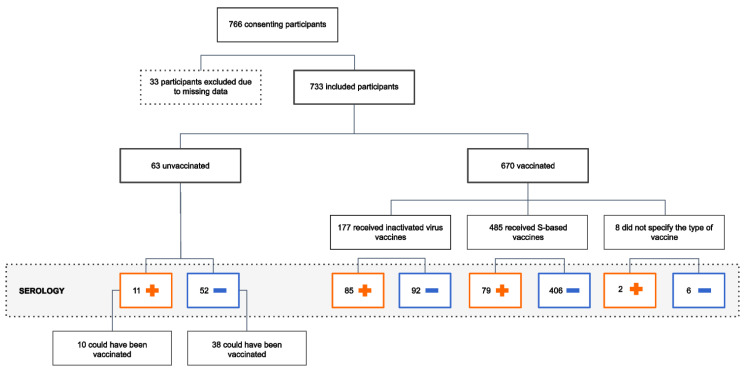
Sampling strategy and serology results for vaccinated and unvaccinated participants. Participants who had missing data and/or nonviable biological samples were excluded from the analysis. The final sample consists of 733 participants, 63 of them were not vaccinated, although 48 could have been.

A total of 766 residents of São Sebastião consented to participate in our study. Among them, 33 participants were excluded from the final analysis due to missing data (e.g., participants who did not complete the questionnaire or whose biological samples were unsuitable for serological analysis). Therefore, our final sample consisted of 733 participants, 670 (91.40%) of whom had been vaccinated at the time of collection (Figure 1).

Out of the 733 participants, 63.98% (469/733) were female. Age ranged from 1 to 93 years old (median age 38; quartiles 28–50), and 77.35% (567/733) were self-declared black or mixed race. Regarding educational level, 30.83% (226/733) were illiterate or did not complete elementary school, whereas 17.46% (128/733) completed elementary school, 37.65% (276/733) completed high school, and 9.41% (69/733) graduated from college or higher. Regarding monthly income, 33.97% (249/733) declared living with up to one minimum wage (BRL 1100.00; USD 216.21 as of January 2023) and 1.64% (12/733) with more than 5 minimum wages (BRL 5500.00; USD 1081.04 as of January 2023). The number of cohabitants per household ranged from 1 to 10 people (average 3.67; median of 4 people—quartiles 3–5). A total of 32.20% (236/733) declared to have at least one chronic disease, including hypertension (48.30%; 114/236) and diabetes. See Table 1 for data related to sociodemographic characteristics and Supplementary Table S1 for data related to comorbidities.

Table S1. Comorbidities declared by participants. Data are shown for the overall sample and vaccinated and unvaccinated participants. Overlapping CIs indicate no discernible variation in seroprevalence between individuals with or without comorbidities regardless of vaccination status.

### 3.2. Vaccinated Participants

**Figure 2:**
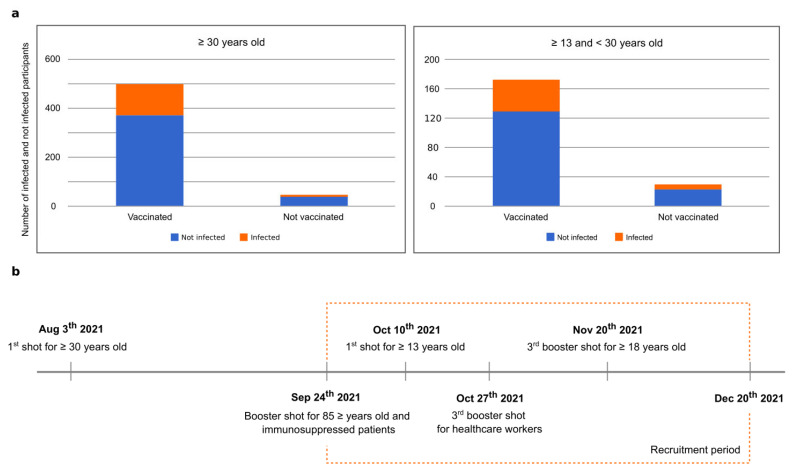
(**a**) Proportion of infected participants among vaccinated and unvaccinated participants for participants ≥30 years old (**left**) and ≥13 and <30 years old (**right**). (**b**) A timeline highlighting the dates when population groups had access to vaccination in São Sebastião and the time frame our survey was conducted.

Out of the 733 participants, 91.40% (670/733) had received at least one COVID-19 vaccine shot at the time of collection. Among them, 26.42% (177/670) received whole inactivated virus vaccines, 72.39% (485/670) received spike (S) protein mRNA vaccines, and 1.19% (8/670) did not specify which vaccine they received (Figure 2). Among vaccinated participants, 72.09% (483/670) were fully vaccinated (i.e., received the second dose of a two-dose series vaccine or one dose of a single-dose vaccine recommended for their age group). It is important to note that heterologous vaccination was not available at the time of our survey.

Among the participants vaccinated with at least one dose, 64.77% (434/670) were female and 77.16% (517/670) were self-declared black or mixed race. Age ranged from 13 to 93 years (median age 42; quartiles 32–54). A total of 30.75% (206/670) were illiterate or did not complete elementary school, 17.61% (118/670) had completed elementary school, 37.91% (254/670) had completed high school, and 9.70% (65/670) graduated from college. A total of 38.50% (228/670) of vaccinated participants declared living with up to one minimum wage per month (BRL 1100.00; USD 216.21 as of January 2023). The total number of cohabitants per household ranged from 1 to 10 people among the vaccinated participants [median number of people 4; (quartiles 3–5)] (Table 1). A total of 33.58% (225/670) of the vaccinated participants reported having a comorbidity, including hypertension (16.27%; 109/670) and diabetes (8.51%; 57/670) (Supplementary Table S1).

### 3.3. Unvaccinated Participants

Out of the 733 participants, 8.59% (63/733) were not vaccinated. Among them, 76.19% (48/63) could have been vaccinated with at least one shot at the time our survey was conducted. The remaining 15 participants were below the age allowed for vaccination in Brazil at the moment of our survey. Among the 48 unvaccinated participants that could have been vaccinated, 50.00% (24/48) were female and their ages ranged from 20 to 69 years old (median age 33.5; quartiles 27.75–45.5). A total of 81.25% (39/48) of them self-declared black or mixed race. A total of 31.25% (15/48) were illiterate or did not complete elementary school, 16.67% (8/48) had completed elementary school, 39.58% (19/48) had completed high school, 6.25% (3/48) graduated from college, and 6.25% (3/48) did not answer the question. Regarding monthly income, 58.33% (28/48) declared living with up to one minimum wage per month (BRL 1100.00; USD 216.21 as of January 2023). The number of cohabitants per household in the unvaccinated participant group ranged from 1 to 8 people with a median of 4 (quartiles 2.5–5) (Table 1). Out of the 48 unvaccinated participants that could have been vaccinated, 16.67% (8/48) reported having a comorbidity including hypertension (6.25%; 3/48), diabetes (4.17%; 2/48), cancer (2.08%; 1/48), depression (2.08%; 1/48), fibromyalgia (2.08%; 1/48), and heart conditions (2.08%; 1/48) (Supplementary Table S1). 

### 3.4. Overall Seroprevalence

The presence of immunoglobulin G (IgG) against the nucleocapsid protein (N) of SARS-CoV-2 was detected in 24.15% (177/733) of the participants. Therefore, the overall estimated seroprevalence of SARS-CoV-2 infection in residents of São Sebastião was 24.15% (95% CI 21.19–27.38; 177/733). Seroprevalence was similar among women and men. We did not find a difference in seroprevalence according to age, self-declared race, educational level, monthly income, or number of cohabitants in households (Table 1). The prevalence ratio (PR) of having a comorbidity was 0.91 (95% CI 0.71–1.17; *p*-value 0.479).

### 3.5. Seroprevalence in Vaccinated Participants

The estimated seroprevalence of IgG against the SARS-CoV-2 N-protein for the 670 vaccinated participants was 24.77% (95% CI 21.50–28.04; 166/670), with a PR of 1.03 (95% CI 0.98–1.08; *p*-value 0.131). Considering only the participants who received an mRNA vaccine with S-based epitope (485 participants), the estimated seroprevalence of IgG against SARS-CoV-2 N-protein decreased to 16.29% (95% CI 13.04–19.85; 79/485), with a PR reduced to 0.59 (95% CI 0.47–0.68; *p*-value < 0.05) (see Table 1).

### 3.6. Seroprevalence in Unvaccinated Participants

The estimated seroprevalence of IgG against the SARS-CoV-2 N-protein among unvaccinated participants was 17.46% (95% CI 10.04–28.62; 11/63). When we consider only the 48 unvaccinated participants that could have already been vaccinated at the time our survey was conducted, the estimated seroprevalence slightly increases to 20.83% (95% CI 11.73–34.26; 10/48). Such variation was not statistically significant, as CIs overlap. Their attributable risk was 0.2788, suggesting that 27.88% of SARS-CoV-2 infections among these participants could have been prevented if they were vaccinated.

## 4. Discussion

Our results show an overall seroprevalence of 24.15% (95% CI 21.19–27.38; 177/733) in São Sebastião and no differences between groups and strata (regarding socioeconomic and demographic characteristics such as sex, age, self-declared race/ethnicity, educational level, monthly income, number of cohabitants, and comorbidities). These results are presented in Table 1 and Table S1, where the overlapping of confidence intervals between groups is shown. Among vaccinated participants (91.40%; 670/733), our estimated seroprevalence was 24.77% (95% CI 21.50–28.04; 166/670), while among the unvaccinated, it was 17.46% (95% CI 10.04–28.62; 11/63). There is, however, a difference in seroprevalence between participants who received whole inactivated virus vaccines (26.42%; 177/670) and those who received S-based vaccines [16.29% (95% CI 13.04–19.85; 79/485)]. This discrepancy between seroprevalence results may be due to the fact that whole inactivated virus vaccines may induce a humoral response against the nucleocapsid protein, which is highly immunogenic [14]. Considering seroprevalence in the unvaccinated group, we calculated an attributable risk of 0.2788, suggesting that 27.88% of SARS-CoV-2 infections among these participants could have been prevented with vaccines based on the S protein.

The overall seroprevalence we found in São Sebastião and the lack of difference between groups and strata differ from what studies in other Brazilian populations found (see [15,16,17,18] for examples). That discrepancy is likely related to the time at which the surveys were conducted, as well as to the characteristics of the populations under scrutiny and to those of the tests used. Our study was conducted between 24 September and 19 December 2021 in a region (Centro-Oeste) that was not as harshly struck by the pandemic as northern, northeastern, and southeastern Brazil [19]. Further, our sample includes a high proportion of vaccinated participants (91.40%; 670/733).

At the time of our survey, 91.40% (670/733) of our participants had received at least one vaccine shot, and 72.09% of these participants were fully vaccinated. Among the 63 (8.59%; 63/733) participants who were not at all vaccinated, 6.55% (48/733) could have received at least one shot of the vaccine. Given that the seroprevalence in the unvaccinated group was 20.83% (95% CI 11.73–34.26; 10/48), 27.88% of COVID-19 cases could have been prevented if they had been vaccinated with S-protein-based vaccines.

Our study included participants who received a whole inactivated virus vaccine (26.42%; 177/670) as well as mRNA-based vaccines based on the S protein (72.39%; 485/670). Overall seroprevalence [24.15% (95% CI 21.19–27.38)] was slightly lower than among vaccinated participants [24.77% (95% CI 21.50–28.04; 166/670)]. Such difference, though not statistically significant as CIs overlap, might be due to the presence of antibodies induced by whole inactivated virus vaccines, which were taken by a significant portion of our sample (26.42%; 177/670). Further, seroprevalence was lower among participants who received S-based vaccines [16.29% (95% CI 13.04–19.85; 79/485)].

The prevalence ratio we found for participants who were vaccinated and carried detectable IgG was 1.03 (95% CI 0.98-–1.08; *p* value 0.131). That alone would indicate that vaccination is not a protective measure; nevertheless, a fraction of our sample received whole inactivated virus vaccines. Combined, those two observations, which are not statistically significant, stem from the fact that differentiating seroconversion derived from vaccination with whole inactivated virus vaccines and from natural infection is not a straightforward task. On the other hand, while the presence of comorbidities is a well-known risk factor for COVID-19 [20], our results (PR for having a comorbidity and detectable IgG: 0.91; 95% CI 0.71–1.17; *p* value 0.479) imply that having a comorbidity would confer a protective effect—which is indeed explained by the high proportion of vaccinated participants in our sample carrying a comorbidity (50/53). What we can, in fact, infer from our results is that vaccination has a protective effect, as there was no difference in seroprevalence between participants with and without comorbidities.

The reasons why those 48 participants were not vaccinated elude us. For a vaccination program to succeed, it must consider a number of factors. It must rely on data regarding the effectiveness of vaccines themselves, the effectiveness of a two-dose course versus an annual dose, the effect of the increasing level of exposure of human populations, and the periodic emergence of new strains. Distribution and access are also of utmost importance. In Brazil, there is a public Unified Health System (Sistema Único de Saúde) holding the structure for the distribution of vaccines and conduction of mass vaccination programs, as well as for year-round vaccination to the entire population [21]. There are also outreach strategies such as the “*carro da vacina*” (a mobile vaccination structure), and the in-home vaccination of people with mobility issues have been in place. Regardless, over the course of the pandemic, we saw unusual vaccine hesitancy, defined as “delay in acceptance or refusal of safe vaccines despite availability of vaccination services” [22].

Vaccine hesitancy is context-specific, including factors as diverse as politics, ethnicity, education level, perception of the disease in question, confidence in the safety and effectiveness of the vaccine [23], and confidence in government and health authorities [24]. Surveys conducted in several countries from both the Global North (Australia, the United Kingdom, and the United States) [23,24,25] and the Global South (Colombia and a series of African and Asian countries) [25] found that the reasons for vaccine hesitancy vary widely and many times contradictorily according to socioeconomic, demographic, political, and health factors. We believe a number of different factors, mostly related to politics and policies, can interfere with vaccination hesitancy and the rate of vaccination in Brazil. In Brazil, COVID-19 vaccination began on 17 January 2021 [21], with the distribution of an imported whole inactivated virus vaccine to priority groups stipulated by the Ministry of Health. The whole inactivated virus vaccine produced in Brazil (Coronavac/Sinovac), which had been developed by Instituto Butantan and could have been under production since late 2020, was only made available to Brazilians at the end of February 2021. Batches of imported viral vector and mRNA-based vaccines arrived in the country by the end of January and May 2021, respectively [21]. Nevertheless, political choices of a far-right inclination led President Jair Bolsonaro to intentionally delay the implementation of vaccination policies [21] and to fuel a strong fake news apparatus when early vaccination could have prevented thousands of deaths. Finally, more practical reasons can have played an important role: the cost of the dislocation to a vaccination point or the impossibility of losing a day of work may have prevented many people from getting vaccinated, for instance.

### Limitations

Over the course of our research, we encountered a few limitations either in execution, methodological choices, and/or intrinsic to cross-sectional studies. First of all, regarding sample size calculations, the data most closely related to our study population were collected by us (data not yet published) in Cidade Estrutural, another city in the Federal District which we have been studying extensively since early 2021. Besides the close proximity, both populations share many sociodemographic characteristics that are relevant to the course of the COVID-19 epidemic in Brazil. Further, at the time our surveys were conducted, São Sebastião and Cidade Estrutural had similar seroprevalences. It is also important to note that the Omicron variant had not yet been detected in Brazil at the time of our survey. While we do have data on the SARS-CoV-2 variants that were circulating in Cidade Estrutural in the timeframe of our survey, we do not have that information for São Sebastião. DELTA was the predominant variant circulating in Cidade Estrutural (data not published) and in Distrito Federal, where both cities are located. Hence, DELTA was very likely predominant in São Sebastião. Because our sample size was calculated solely to obtain reliable estimations of seroprevalence, it is unfortunately small for some other analyses. It does not include a sufficient number of unvaccinated participants for stratified analysis within this group, for instance.

Regarding georeferencing, due to setbacks in data collection, we were not able to investigate the spatial distribution of seroprevalence. Nevertheless, because our primary data do not differ from official data [11], we believe our sample is indeed representative of the population.

Our sample was composed of participants who received mRNA-based vaccines based on the S protein and/or on whole inactivated viruses. Whole inactivated virus vaccines can induce the production of antibodies against a number of targets, which in this case include the nucleocapsid protein [5], and thus may falsely increase the estimation of overall seroprevalence. Because the S protein is the primary target for neutralizing antibodies induced by either vaccines or natural infection, the detection of anti-N-protein antibodies could, in theory, be used as a proxy for the detection of natural infection in people who received S-protein-based vaccines [26]. We cannot, however, distinguish whether the source of the anti-N-protein IgG is a natural infection or vaccination in people who received whole inactivated virus vaccines. It is important to note that Fonseca et al. [27] found that one of their participants who had received whole inactivated virus vaccines tested negative for anti-N-protein IgG, but positive for anti-S-protein. Hence, our seroprevalence estimates might actually reflect natural infection rather than vaccination. Also, because IgG titers decline over time, seronegative participants whose titers fell below a certain threshold could have had a previous infection that went unnoticed. That does not however affect seroprevalence estimates, as they are measured in terms of detectable antibodies.

Test sensitivity and specificity can also impact seroprevalence estimates. However, we do not believe that to be the case in our study, since the test we used (anti-SARS-CoV-2 IgG chemiluminescent microparticle immunoassay (CMIA)) has both high sensitivity and specificity [81.8 (74.7–87.3%), 99.3 (96.3–99.9%) or higher after 15 days since infection [28]]. We do acknowledge that different methodologies may yield different results and that comprehending what stems from methodological approaches and what results from the actual dynamics of the disease in a given population, therefore, depends on studies that are, in fact, comparable [29].

## 5. Conclusions

Our work has centered on the seroprevalence of IgG against the SARS-CoV-2 N-protein. We described seroprevalence in the overall population, as well as in vaccinated and unvaccinated subgroups. Our observations led to the conclusion that the detection of anti-N-protein IgG could, in theory, be used as a proxy for the detection of natural infection in people who received S-protein-based vaccines. Differentiating humoral response derived from natural infection and from vaccination is not a simple endeavor but might be feasible after all. The task would definitely benefit from the analysis of more than one biomarker, i.e., tests for the detection of both anti-N- and anti-S-protein IgG in conjunction with information on individual vaccination.

Lastly, we were only able to speculate about vaccine hesitancy, since we did not collect the data necessary for analysis, and, even if we had, 91.40% (670/733) of our sample was vaccinated. In spite of the political climate and the other factors we listed as possible causes for vaccine hesitancy, only 48 out of our 733 participants had not been vaccinated, even though they could have been. Further, only 8 out of the 236 (0.3%; 6/236) participants who declared having a comorbidity had not been vaccinated. We believe, therefore, that the Brazilian positive culture towards vaccination might have overcome the disinformation and the consequent hesitancy that could have led to lower vaccination coverage after all. Inquiries on the reasons for vaccine hesitancy might help direct vaccination efforts and help target the groups more prone to hesitancy, as it has been shown to be related to any vaccination and not only against COVID-19.

## Figures and Tables

**Table 1:** Seroprevalence according to sociodemographic characteristics. Data are shown for the overall sample and for vaccinated and unvaccinated participants. Overlapping CIs indicate no discernible variation in seroprevalence between groups and strata.

	Overall Sample n = 733			Vaccinated Participants n = 670 (91.41%)			Unvaccinated Participants n = 63 (8.59%)		
	Positive n = 177 (24.15%)	Negative n = 556 (75.85%)	Prevalence (95% CI)	Positive n = 166 (24.78%)	Negative n = 504 (75.22%)	Prevalence (95% CI)	Positive n = 11 (17.46%)	Negative n = 52 (82.54%)	Prevalence (95% CI)
Sex									
Female	118 (66.67%)	351 (63.13%)	25.16 (21.21–29.09)	112 (67.47%)	322 (63.89%)	25.81 (21.69–29.92)	6 (54.55%)	29 (55.77%)	17.14 (4.66–29.63)
Male	58 (32.77%)	201 (36.15%)	22.39 (17.32–27.47)	53 (31.93%)	179 (35.52%)	22.84 (17.44–28.25)	5 (45.45%)	22 (42.31%)	18.52 (3.87–33.17)
Did not answer	1 (0.56%)	1 (0.18%)	-	1 (0.60%)	3 (0.60%)	-	0 (0.0%)	1 (1.92%)	-
Age (years)									
1–10	0 (0.0%)	2 (0.36%)	-	0 (0.0%)	0 (0.0%)	-	0 (0.0%)	2 (3.85%)	-
11–20	12 (6.78%)	42 (7.55%)	22.22 (11.13–33.31)	9 (5.42%)	36 (7.14%)	20.00 (8.31–31.69)	3 (27.27%)	6 (11.54%)	33.33 (2.53–64.13)
21–30	38 (21.47%)	125 (22.48%)	23.31 (16.82–29.80)	36 (21.69%)	110 (21.82%)	24.66 (17.67–31.65)	2 (18.18%)	15 (28.85%)	11.76 (1–27.08)
31–40	50 (28.25%)	136 (24.46%)	26.88 (20.51–33.25)	46 (27.71%)	126 (25.00%)	26.74 (20.13–33.36)	4 (36.36%)	10 (19.23%)	28.57 (4.91–52.24)
41–50	37 (20.91%)	117 (21.05%)	24.03 (17.28–30.77)	36 (21.69%)	110 (21.82%)	24.66 (17.67–31.65)	1 (9.09%)	7 (13.64%)	12.50 (1–35.42)
51–60	20 (11.30%)	80 (14.39%)	20.00 (12.16–27.84)	19 (11.45%)	76 (15.08%)	20.00 (11.96–28.04)	1 (9.09%)	4 (7.69%)	20.00 (1–55.06)
61–70	16 (9.04%)	39 (7.01%)	29.09 (17.09–41.09)	16 (9.64%)	32 (6.35%)	33.33 (20.00–46.67)	0 (0.0%)	7 (13.64%)	-
71–80	2 (1.13%)	7 (1.26%)	22.22 (0–49.38)	2 (1.20%)	7 (1.39%)	22.22 (1–49.38)	0 (0.0%)	0 (0.0%)	-
≥81	1 (0.56%)	6 (1.08%)	14.29 (0–40.21)	1 (0.60%)	6 (1.20%)	14.29 (1–40.21)	0 (0.0%)	0 (0.0%)	-
Did not answer	1 (0.56%)	2 (0.36%)	-	1 (0.60%)	1 (0.20%)	-	0 (0.0%)	1 (1.92%)	-
Self-declared race									
Black or mixed race	133 (75.14%)	434 (78.06%)	23.45 (19.97–26.94)	124 (74.70%)	393 (77.98%)	23.98 (20.30–27.66)	9 (81.82%)	41 (78.85%)	18.00 (7.35–28.65)
Other races	37 (20.91%)	106 (19.06%)	25.87 (18.70–33.05)	36 (21.69%)	96 (19.04%)	27.27 (19.67–34.87)	1 (9.09%)	10 (19.23%)	9.09 (1–26.08)
Did not answer	7 (3.95%)	16 (2.88%)	-	6 (3.61%)	15 (2.98%)	-	1 (9.09%)	1 (1.92%)	50.00 (1–100.00)
Education									
Illiterate or incomplete elementary school	52 (29.38%)	174 (31.30%)	23.01 (17.52–28.50)	50 (30.12%)	156 (30.95%)	24.27 (18.42–30.13)	2 (18.18%)	18 (34.62%)	10.00 (1–23.15)
Elementary school	32 (18.08%)	96 (17.26%)	25.00 (17.50–32.50)	30 (18.07%)	88 (17.46%)	25.42 (17.57–33.28)	2 (18.18%)	8 (15.38%)	20.00 (1–44.79)
High school	66 (37.29%)	210 (37.77%)	23.91 (18.88–28.95)	62 (37.35%)	192 (38.10%)	24.41 (19.13–29.69)	4 (36.36%)	18 (34.62%)	18.18 (2.06–34.30)
College	16 (9.04%)	53 (9.53%)	23.18 (13.23–33.15)	16 (9.64%)	49 (9.72%)	24.62 (14.14–35.09)	0 (0.0%)	4 (7.69%)	-
Did not answer	11 (6.21%)	23 (4.14%)	-	8 (4.82%)	19 (3.77%)	-	3 (27.27%)	4 (7.69%)	42.86 (6.20–79.52)
Monthly income									
Up to 1 minimum wage	54 (30.51%)	195 (35.05%)	21.69 (16.57–26.81)	50 (30.12%)	178 (35.32%)	21.93 (16.56–27.30)	4 (36.36%)	17 (32.69%)	19.05 (2.25–35.84)
2–3 minimum wages	63 (35.59%)	164 (29.50%)	27.75 (21.93–33.58)	62 (37.35%)	154 (30.56%)	28.70 (22.67–34.74)	1 (9.09%)	10 (19.23%)	9.09 (1–26.08)
4–5 minimum wages	9 (5.08%)	29 (5.22%)	23.68 (10.16–37.20)	9 (5.42%)	27 (5.36%)	25.00 (10.85–39.15)	1 (9.09%)	2 (3.85%)	33.33 (1–86.68)
≥5 min wages	4 (2.26%)	8 (1.44%)	3.33 (0.66–60.01)	3 (1.81%)	8 (1.59%)	27.27 (9.50–53.59)	0 (0.0%)	0 (0.0%)	-
Did not answer	47 (26.55%)	160 (28.78%)	-	42 (25.30%)	137 (27.18%)	-	5 (45.45%)	23 (44.23%)	17.86 (3.67–32.04)
Number of cohabitants									
1–2	26 (14.69%)	136 (24.46%)	16.05 (10.40–21.70)	25 (15.06%)	123 (24.40%)	16.89 (10.85–22.92)	1 (9.09%)	13 (25.00%)	7.14 (1–20.63)
3–4	89 (50.28%)	275 (49.46%)	24.45 (20.04–28.87)	85 (51.20%)	254 (50.40%)	25.07 (20.45–29.69)	4 (36.36%)	21 (40.38%)	16.00 (1.63–30.71)
5–6	45 (25.42%)	106 (19.06%)	29.80 (22.51–37.10)	40 (24.10%)	94 (18.65%)	29.85 (22.10–37.60)	5 (45.45%)	12 (23.08%)	29.41 (7.75–51.07)
7–8	8 (4.52%)	23 (4.14%)	25.81 (10.40–41.21)	8 (4.82%)	20 (3.97%)	28.57 (11.84–45.30)	0 (0.0%)	3 (5.77%)	-
9–10	2 (1.13%)	4 (0.72%)	0.33 (0–17.05)	2 (1.20%)	4 (0.79%)	33.33 (0–71.0)	0 (0.0%)	0 (0.0%)	-
Did not answer	7 (3.95%)	12 (2.16%)	-	6 (3.61%)	9 (1.79%)	-	1 (9.09%)	3 (5.77%)	-
Reported a comorbidity	53 (29.94%)	183 (32.92%)	22.46 (17.13–27.78)	50 (30.12%)	175 (34.72%)	22.22 (16.79–27.65)	3 (27.27%)	8 (15.38%)	27.27 (9.50–53.59)

## Data Availability

Data is available upon request to the email carolinacarvalhogontijo@gmail.com.

## Supplementary Materials

The following supporting information can be downloaded at: https://www.mdpi.com/article/10.3390/vaccines11050916/s1, Figure S1. Flowchart of participant recruitment. Randomly selected GPS points were visited by field teams. In case the point fell on a residential lot and there was at least one resident present, all residents were invited to participate. One was randomly selected using a random numbers table, and, after signing a consent form, was interviewed and had biological samples collected. Checklist S1. Checklist for the Strengthening the Reporting of Observational Studies in Epidemiology (STROBE) guidelines. Supplementary Table S1. Comorbidities declared by participants. Results are shown for vaccinated and unvaccinated participants and for the overall sample.
