# Supplementary material for: Seroprevalence of SARS-CoV-2 and Vaccination Coverage among Residents of a Lower-Middle-Class Population in the Federal District, Brazil
Source: Vaccines (Basel). 2023 Apr 28;11(5):916. doi: 10.3390/vaccines11050916 (PMC10220777; doi:10.3390/vaccines11050916)
Supplement: Supplementary file 1 [file vaccines-11-00916-s001.zip › supplementary-materials/supplementary-titles-legends.docx]

**Figure S1.** Flowchart of participant recruitment. Randomly selected GPS points were visited by field teams. In case the point fell on a residential lot and the was at least one resident present, all residents were invited to participate. One was randomly selected using a random numbers table, and after signing a consent form, was interviewed and had biological samples collected.

**Checklist S1.** Checklist for the Strengthening the Reporting of Observational Studies in Epidemiology (STROBE) guidelines.

**Supplementary Table S1.** Comorbidities declared by participants. Results are shown for vaccinated and unvaccintaed participants, and for the overall sample.
