# Supplementary material for: Seroprevalence of SARS-CoV-2 and Vaccination Coverage among Residents of a Lower-Middle-Class Population in the Federal District, Brazil
Source: Vaccines (Basel). 2023 Apr 28;11(5):916. doi: 10.3390/vaccines11050916 (PMC10220777; doi:10.3390/vaccines11050916)
Supplement: Supplementary file 1 [file vaccines-11-00916-s001.zip › supplementary-materials/tableS1.docx]

**Supplementary Table S1.** Comorbidities declared by participants. Results are shown for vaccinated and unvaccinated participants, and for the overall sample. Overlapping CIs indicate no discernible variation in seroprevalence between individuals with or without comorbidities regardless of vaccination status.

|  | Vaccinated  (n=670) | | | Unvaccinated  (n=63) | | | Overall sample  (n=733) | |
| --- | --- | --- | --- | --- | --- | --- | --- | --- |
|  | Positive  (n = 166) | Negative (n=504) | Prevalence | Positive  (n = 11) | Negative  (n= 52) | Prevalence | Overall  (n=733) | Prevalence |
| Hypertension | 24 | 85 | 22.02  (14.24-29.80) | 1 | 5 | 16.67  (0-46.49) | 115 | 21.74  (14.20-29.28) |
| Diabetes | 10 | 47 | 17.54  (7.67-27.42) | 0 | 3 | - | 60 | 16.67  (7.24-26.10) |
| Heart conditions (angina, mitral valve disease, arrhythmia, heart murmur) | 1 | 10 | 9.09  (0-26.08) | 0 | 0 | - | 11 | 9.09  (0-26.08) |
| Endocrine disorders (i.e. hypothyroidism, hyperthyroidism) | 3 | 7 | 30.00  (0.01-58.54) | 1 | 0 | - | 11 | 36.36  (7.94-64.79) |
| Psychiatric disorders (i.e. depression, anxiety) | 4 | 7 | 36.36  (7.94-64.79) | 1 | 0 | - | 11 | 45.45  (16.03-74.88) |
| Neurological disorders (i.e. fibro-myalgia, epilepsy) | 2 | 6 | 25.00  (0-55.01) | 0 | 1 | - | 9 | 22.22  (0-49.38) |
| Rhinitis and Sinusitis | 1 | 7 | 12.50  (0-35.42) | 0 | 0 | - | 8 | 12.50  (0-35.42) |
| Bronchitis | 1 | 7 | 12.50  (0-35.42) | 0 | 0 | - | 8 | 12.50  (0-35.42) |
| Pulmonary disease | 1 | 6 | 14.29  (0-40.21) | 0 | 0 | - | 7 | 14.29  (0-40.21) |
| Kidney disease | 0 | 7 | - | 0 | 0 | - | 7 | - |
| High cholesterol | 2 | 3 | 40.00  (0-82.94) | 0 | 0 | - | 5 | 40.00  (0-82.94) |
| Cancer | 2 | 2 | 50.00  (1.00-99.00) | 0 | 0 | - | 4 | 50.00  (1.00-99.00) |
| Thrombosis | 0 | 3 | - | 0 | 0 | - | 3 | - |
| Chagas disease | 1 | 2 | 33.33  (0-86.68) | 0 | 0 | - | 3 | 33.33  (0-86.68) |
| Osteoporosis | 2 | 1 | 66.67  (13.32-100) | 0 | 0 | - | 3 | 66.67  (13.32-100) |
| HIV | 1 | 1 | 50.00  (0-100) | 0 | 0 | - | 2 | 50.00  (0-100) |
| Lupus | 0 | 2 | - | 0 | 0 | - | 2 | - |
| Anemia | 0 | 2 | - | 0 | 0 | - | 2 | - |
| Herniated disc | 1 | 1 | 50.00  (0-100) | 0 | 0 | - | 2 | 50.00  (0-100) |
| Liver disease | 0 | 1 | - | 0 | 0 | - | 1 | - |
| Diverticulitis | 0 | 1 | - | 0 | 0 | - | 1 | - |
| Lactose intolerance | 0 | 0 | - | 0 | 1 | - | 1 | - |
| Endometriosis | 0 | 1 | - | 0 | 0 | - | 1 | - |
